# Congenital Renal Fusion and Ectopia in the Trauma Patient

**DOI:** 10.1155/2016/5203872

**Published:** 2016-11-08

**Authors:** Andrew A. Rosenthal, Jordan J. Ditchek, Seong K. Lee, Rafael Sanchez, Chauniqua Kiffin, Dafney L. Davare, Eddy H. Carrillo

**Affiliations:** ^1^Division of Acute Care Surgery and Trauma, Memorial Regional Hospital, 3501 Johnson Street, Hollywood, FL 33021, USA; ^2^Department of Radiology, Memorial Regional Hospital, 3501 Johnson Street, Hollywood, FL 33021, USA

## Abstract

We present two separate cases of young male patients with congenital kidney anomalies (horseshoe and crossed fused renal ectopia) identified following blunt abdominal trauma. Despite being rare, ectopic and fusion anomalies of the kidneys are occasionally noted in a trauma patient during imaging or upon exploration of the abdomen. Incidental renal findings may influence the management of traumatic injuries to preserve and protect the patient's renal function. Renal anomalies may be asymptomatic or present with hematuria, flank or abdominal pain, hypotension, or shock, even following minor blunt trauma or low velocity impact. It is important for the trauma clinician to recognize that this group of congenital anomalies may contribute to unusual symptoms such as gross hematuria after minor trauma, are readily identifiable during CT imaging, and may affect operative management. These patients should be informed of their anatomical findings and encouraged to return for long-term follow-up.

## 1. Introduction

Despite being rare, ectopic and fusion anomalies of the kidneys are occasionally noted in a trauma patient during imaging or upon exploration. Today, these anomalies are usually noted as incidental findings during the initial workup with ultrasound or computed tomography (CT scan). In the past, they were identified with the use of intravenous pyelography. These variations in renal anatomy also may be discovered in the operating theater during laparotomy or upon exploration of the retroperitoneum [1]. The presence of such anomalies influences the management of traumatic injuries to the entire renal collecting system and can significantly alter management of a patient's overall renal function. We discuss two such cases of anomalous fusion and ectopy and review the relevance of these congenital anomalies in a trauma setting.

## 2. Case Presentation

### 2.1. Case 1: Horseshoe Kidney

**Figure 1:**
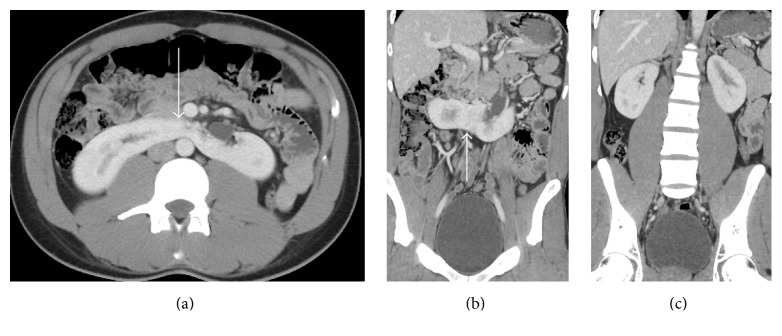
27-year-old male with horseshoe kidney. Axial (a) and coronal (b) contrast-enhanced CT images show the inferior aspect of a horseshoe kidney in the lower abdomen. Note the enhancing isthmus of renal parenchyma crossing the midline (arrow). A coronal contrast-enhanced CT image (c) obtained more posteriorly shows the upper poles of the horseshoe kidney in the renal fossae.

An unrestrained 27-year-old male presented following a motor vehicle crash with blunt abdominal trauma and closed head injury, with Abbreviated Injury Score (AIS) and Injury Severity Score (ISS) of one (minor). His initial exam was relevant for decreased sensorium and mild abdominal tenderness. Vital signs were normal and a Focused Abdominal Sonogram for Trauma (FAST) was of limited value due to poor visualization of the kidney-liver and kidney-spleen interface [2]. Subsequent CT imaging was performed and an uninjured horseshoe kidney was identified (Figures 1(a), 1(b), and 1(c)). All laboratory work including urinalysis was normal. No intervention was required, the patient was informed of his anatomical variations, and the subsequent hospital course was uneventful.

### 2.2. Case 2: Crossed Renal Ectopy without Fusion

A healthy 31-year-old male presented after suffering blunt abdominal trauma during a soccer match. He presented with right-sided abdominal pain and gross hematuria, with AIS of two and ISS of 12 (moderate). Vital signs were normal and exam was significant only for right-sided flank and abdominal pain. The FAST exam was abnormal as the left kidney was not visualized and the right kidney-liver interface was noted to be distorted and oriented in an unusual plane. Gross hematuria was noted upon bladder catheterization.

**Figure 2:**
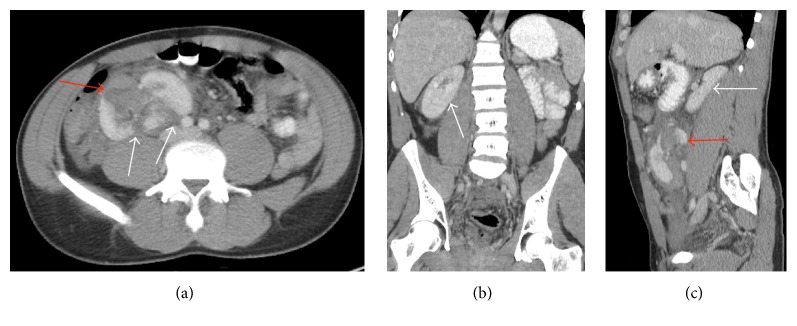
31-year-old male with crossed unfused renal ectopia. Axial contrast-enhanced CT image (a) shows lacerations (white arrows) through the posterior cortex of the ectopic left kidney, positioned anteriorly and inferiorly in the right abdomen. A perinephric hematoma (red arrow) is noted anteriorly. A coronal CT image (b) shows the uninjured right kidney in a normal location. A sagittal CT image (c) through the right abdomen shows the right kidney (white arrow) in a normal location in the renal fossa and the ectopic kidney (red arrow) in the lower right abdomen. Note the gap between these kidneys in this unfused ectopia.

A CT was done demonstrating crossed unfused renal ectopia with two right-sided kidneys and an absent left kidney, with laceration injury to the more anterior and inferior right-sided kidney, and hemoperitoneum in multiple abdominal quadrants (Figures 2(a), 2(b), and 2(c)). HGB was 12.5 g/dL, and creatinine level was normal.

The patient was admitted for hydration and observation, with serial exams. Hematuria resolved with intravenous hydration and bedrest over 48 hours. Activity and diet were then advanced and the subsequent hospital course was unremarkable. Patient was lost to follow-up; therefore, plans for follow-up evaluation and possible imaging are still pending.

## 3. Discussion

Horseshoe kidney is the most common fusion abnormality with a reported incidence of 1 : 400 and is more common in males [3, 4]. Usually, it is found in a lower lumbar position than normal kidneys, due to the arrest of ascent of the fetal kidney by the inferior mesenteric artery. Since the horseshoe kidney sits directly in front of the lumbar spine without chest wall protection, it may be more easily compressed and injured in blunt abdominal trauma. Even when the finding is incidental, the patient must be educated on its presence due to an increased incidence of urinary infection, pyelonephritis, nephrolithiasis, and both benign and malignant tumors. An association with chromosomal abnormalities such as trisomy 21, 18, and 13 is also well documented [4].

**Figure 3:**
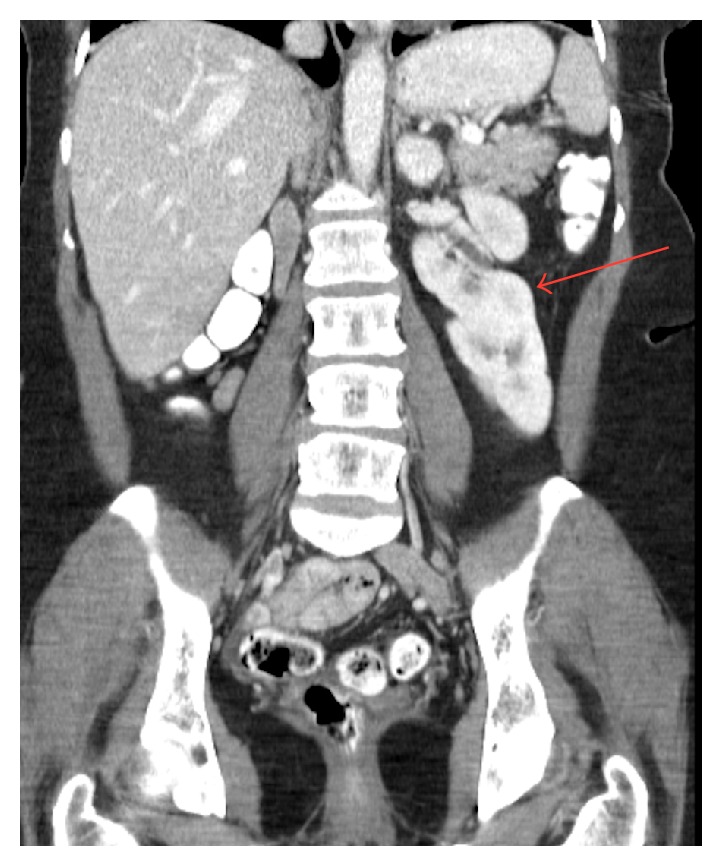
A coronal contrast-enhanced CT image shows an ectopic right kidney fused to the lower pole of the left kidney.

Crossed fused (Figure 3) and unfused renal ectopia is usually identified incidentally during trauma or imaging done for other reasons, often antenatally. Its incidence is roughly 1 : 2000 and also has an increased incidence of infection, obstruction, and stone formation [5]. Like horseshoe kidney, crossed renal ectopia is more common in males [4, 5]. In renal ectopia, the left kidney is frequently crossed to the right side, as presented in case two [5]. Ectopic kidneys often have decreased function, as measured by scintigraphy. While the kidney and ureter cross the midline, the ureteral insertion into the bladder is normal.

**Figure 4:**
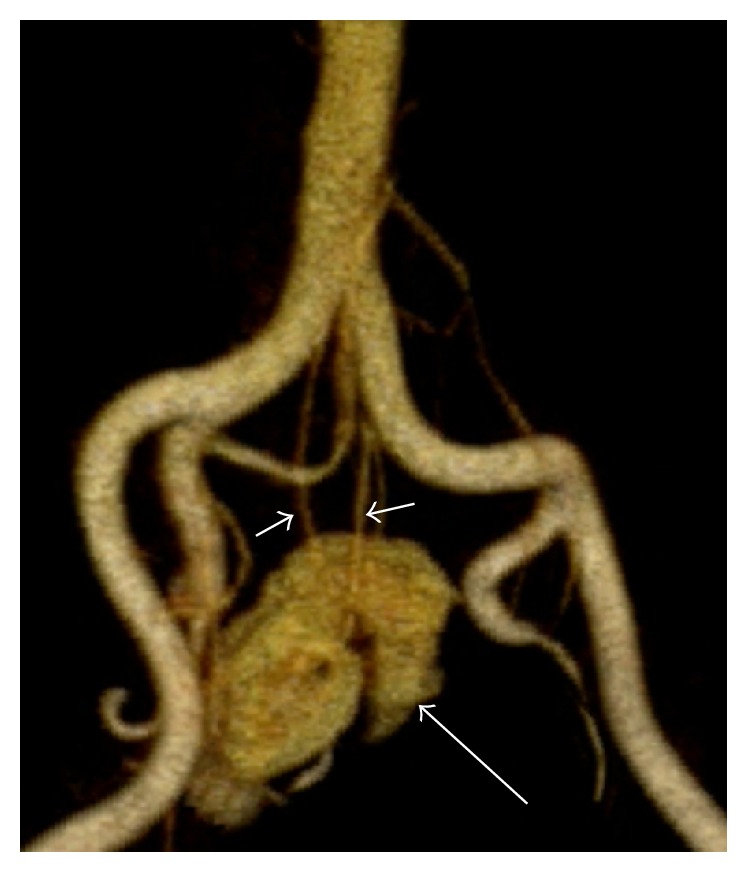
Pelvic kidney. 3D volume-rendered MRA image shows a pelvic kidney (long arrow) positioned in the midline below the aortic bifurcation. Note the variant vascular supply, including multiple anomalous accessory renal arteries (short arrows) arising from the common iliac arteries.

Positional (ectopia) and fusion (horseshoe) renal anomalies occur when there is disruption or deviation in the normal course of fetal renal migration from the pelvis to the ipsilateral respective retroperitoneal renal fossa. Disruption of the normal embryologic migration of the kidney can result in the classic horseshoe variation of renal fusion or can result in a variety of renal anomalies, such as a pelvic kidney or kidney with a crossed (contralateral) location [3, 4]. Trauma practitioners should be aware of these anomalies as their incidental presence at the time of injury may contribute to the patient's presentation; symptoms and findings may alter management. FAST is routinely used in trauma to identify free fluid in the abdomen by visualizing the planes between the intraperitoneal solid organs and the kidneys [2]. An empty renal fossa should direct the ultrasonographer to search for a horseshoe or ectopic kidney. Additionally, surgeons should be aware of the associated variant vasculature that may be present to avoid iatrogenic injury to these vessels in the emergency operative setting (Figure 4).

Urinalysis and culture should be considered to identify traumatic hematuria as well as assess for underlying infection to which these patients are predisposed [4]. Although these congenital abnormalities are usually asymptomatic, they may be associated with urinary tract infection, renal calculi formation, and urinary obstruction, all of which may be existing conditions present at the time of admission for a traumatic injury. A palpable mass may be present on exam.

Since the management of blunt renal trauma is usually nonoperative, the majority of injuries to ectopic and fused kidneys can also be managed without surgical intervention [6]. Minor renal trauma such as contusions, minor disruptions of the fornices, or minor lacerations can be managed nonoperatively with fluid resuscitation alone; major parenchymal disruptions causing hemorrhage or collecting system rupture may require interventional radiology or surgery depending on the patient's clinical presentation. Abnormal kidneys may be more prone to injury when they are more exposed in an anterior position in the abdomen or when they have tumors, cysts, or hydronephrosis. A study by Schmidlin et al. confirmed that patients with abnormal kidneys have lower ISS scores when compared to patients presenting with normal kidneys, and injuries were predominately caused by low impact velocities (<15 km/h) [7].

## 4. Conclusion

A spectrum of renal anomalies exist in the general population and these anatomical variations may impact clinical findings and management. Diseased kidneys are often more susceptible to trauma and patients may present with significant injuries regardless of the mechanism of injury or impact of trauma. Quantifying injury severity using AIS and ISS scores may not include preexisting disease and resulting signs and symptoms. Patients may be asymptomatic or may present with hematuria, flank or abdominal pain, hypotension, or shock. Early abdominal ultrasound may suggest renal abnormality when the kidney is absent or has distorted architecture or unusual orientation. The trauma clinician should consider horseshoe kidney or ectopic/pelvic kidney when the kidney is not found in its usual retroperitoneal position and should consider underlying renal disease or abnormality, like cyst or tumor, when gross hematuria is present after relatively minor trauma.

Horseshoe (fused) kidney and renal ectopy (positional aberrance) occur when the developing kidney has abnormal embryologic and fetal migration and may alter presentation and management. This group of congenital anomalies may contribute to unusual symptoms such as gross hematuria after minor trauma, are easily identifiable during CT imaging, and may affect operative management. Lastly, the patient should always be informed of their anatomy and referred for appropriate follow-up even if the findings are incidental.
